# Editorial: Advancing research, clinical recognition, and targeted therapies for WHIM syndrome

**DOI:** 10.3389/fimmu.2025.1741502

**Published:** 2025-11-27

**Authors:** Christoph B. Geier

**Affiliations:** 1 Division of Immunology, Faculty of Medicine and Health Sciences, University Medicine Oldenburg, Oldenburg, Germany; 2 Institute of Medical Genetics, Faculty of Medicine and Health Sciences, University Medicine Oldenburg, Oldenburg, Germany

**Keywords:** WHIM, CXCR4, CXCR2, CXCR4 antagonists, primary immunodeficiency, leukocyte trafficking, precision medicine

WHIM syndrome, classically defined by warts, hypogammaglobulinemia, infections, and myelokathexis, has evolved from a clinicopathologic curiosity to a mechanistically tractable disorder at the crossroads of chemokine biology, leukocyte trafficking, and precision therapeutics (1–4). This Research Topic assembles six contributions that, taken together, refine the molecular framework of CXCR4 dysregulation, expand variant interpretation and clinical phenotyping, and provide preclinical evidence that informs current and future targeted therapy.

A comprehensive review by Rodríguez-Frade et al. synthesizes the increasingly complex biology of *CXCR4* mutations in WHIM syndrome. Beyond the canonical paradigm of C-terminal truncations that impair receptor phosphorylation, β-arrestin engagement, and ligand-induced internalization, the authors detail how mutant and wild-type CXCR4 differ in quaternary organization, membrane nanoclustering, and coupling to multiple G-protein pathways. These biophysical and signaling nuances help explain why distinct *CXCR4* variants can converge on similar cellular phenotypes (enhanced CXCL12 responsiveness and bone-marrow retention) yet differ in immunologic expression across lineages and tissues. Importantly, the review connects mechanism to therapy, contextualizing the clinical activity of CXCR4 antagonists and highlighting emerging avenues such as gene editing as conceptually disease-modifying approaches.

Complementing this systems view, Zmajkovicova et al. provide a focused mini-review of the expanding *CXCR4* variant landscape and a pragmatic framework for interpretation. With C-terminal variants still comprising the majority of pathogenic mutations in WHIM syndrome, the authors emphasize the limitations of annotation databases and the decisive role of functional assays, particularly standardized internalization, chemotaxis, and ERK/AKT readouts, in reclassifying variants of uncertain significance toward clinical actionability. The review also situates “WHIM-like” states caused by perturbations outside CXCR4, including GRK3 dysfunction and CXCR2 deficiency, reinforcing the diagnostic value of a pathway-centric lens when classical tetrad features are incomplete.

Two original clinical investigations underscore phenotypic heterogeneity and the importance of multilocus thinking. Huang et al. describe a Chinese family with a *CXCR4* V340fs variant producing atypically mild courses relative to the common R334X allele. Despite demonstrable gain-of-function (impaired downregulation, augmented CXCL12 signaling), carriers displayed fewer HPV-related manifestations and complications, with some maintaining intact antibody responses. Immunophenotyping highlighted consistent CD8^+^ T-cell deficiency and altered B-cell compartments. These data refine genotype–phenotype expectations and argue for individualized monitoring strategies that are not solely anchored to the presence or absence of the full WHIM tetrad.

Yilmaz et al. further extend the clinical spectrum with a family harboring a novel *CXCR4* missense variant (p.S341Y) located two residues proximal to E343 and a pathogenic frameshift mutation in *NFKB1*. Functional studies demonstrate that p.S341Y confers partial gain-of-function: increased chemotaxis and ERK/AKT activation with largely preserved internalization, a profile distinct from classical truncations. The proband’s blended WHIM/CVID phenotype, myelokathexis with B-cell maturation arrest and immune dysregulation, emerged only with co-inheritance of NFKB1 haploinsufficiency, while relatives carrying a single variant showed attenuated manifestations. This case illustrates multilocus pathogenic variation as a tangible determinant of clinical expressivity, and it operationalizes the mini-review’s recommendation to pair genetic testing with functional validation when adjudicating causality and tailoring therapy.

The bench-to-bedside arc of CXCR4 antagonism is delineated by two preclinical studies that interrogate distinct, clinically relevant contexts. Roland et al. examine chronic oral CXCR4 inhibition in *Cxcr^4^*+/1013 WHIM knock-in mice. Treatment corrected pan-leukopenia without disrupting granulopoiesis, mobilized functionally competent neutrophils (preserved phagocytosis and oxidative burst), and progressively normalized secondary lymphoid organ architecture, restoring splenic CD8^+^ T-cell numbers and CD4/CD8 ratios, and rebalancing follicular and marginal zone B-cell compartments. These findings extend clinical observations with mavorixafor by providing mechanistic tissue-level correlates and by distinguishing pharmacologic profiles (e.g., longer-acting small molecules versus shorter-acting agents) that may be relevant to durability of immunologic correction.

Nguyen et al. address a long-standing translational question: can targeting the CXCR4 axis benefit neutropenias driven by impaired CXCR2 signaling? Using a pharmacologic *CXCR2* loss-of-function mouse model that recapitulates key patient features (peripheral neutropenia, bone marrow neutrophil retention with myelokathexis-like morphology, and increased susceptibility to pneumococcal pneumonia), the authors demonstrate that chronic CXCR4 antagonism normalizes circulating neutrophil counts, reverses bone marrow sequestration and reduces myelokathexis. Importantly, treatment also improves infection outcomes by restoring neutrophil recruitment to infected lung tissue and lowering bacterial burden and mortality. Together with preclinical and pharmacologic evidence that G-CSF–mediated neutrophil mobilization depends on intact CXCR2 signaling and may be attenuated when this pathway is impaired, these results rationalize clinical exploration of CXCR4 antagonists in CXCR2-deficient chronic neutropenia and in subsets where defective egress, rather than granulopoiesis, underlies cytopenia.

Viewed as a whole, the Research Topic advances three important themes in WHIM syndrome. First, at the level of mechanism, WHIM pathogenesis reflects graded, mutation-specific alterations in receptor regulation and network-level integration that map onto lineage-specific trafficking defects. This mechanistic pluralism might explain clinical variability and underscores why a single biomarker rarely captures disease activity across compartments.

Second, in clinical recognition, both the V340fs family and the S341Y/NFKB1 pedigree emphasize that the absence of the full tetrad does not exclude WHIM, CD8^+^ lymphopenia and specific B-cell subset alterations can be informative even with normal total Ig levels, and multilocus pathogenic variation can decisively shape phenotype. A practical implication is to pair early genetic testing with functional assays when variant classification is uncertain, and to maintain a low threshold for family studies and longitudinal immunophenotyping.

Third, for targeted therapy, convergent preclinical evidence supports CXCR4 antagonism as a mechanism-directed approach that mobilizes effector cells without compromising core functions, with tissue-level benefits that extend beyond transient leukocytosis. The data also open testable hypotheses: optimizing dosing around time-above-threshold metrics for neutrophils and lymphocytes; studying HPV disease modification as a function of tissue trafficking; and evaluating CXCR4 blockade in WHIM-like neutropenias such as CXCR2 deficiency. Parallel progress in curative strategies (e.g., gene editing) warrants coordinated natural-history and outcomes registries to benchmark long-term safety and efficacy across modalities.

In sum, this Research Topic integrates structural and signaling insights, refined diagnostic pathways, and rigorous preclinical pharmacology to move WHIM syndrome further into the realm of precision immunology.
